# Supplementary material for: Role and mechanism of NCAPD3 in promoting malignant behaviors in gastric cancer
Source: Front Pharmacol. 2024 Apr 22;15:1341039. doi: 10.3389/fphar.2024.1341039 (PMC11070777; doi:10.3389/fphar.2024.1341039)
Supplement: Supplementary file 11 [file DataSheet2.ZIP › GSEA/immunologic/my_analysis.Gsea.1599462003817/GSE17974_CTRL_VS_ACT_IL4_AND_ANTI_IL12_12H_CD4_TCELL_UP.html]

Details for gene set GSE17974\_CTRL\_VS\_ACT\_IL4\_AND\_ANTI\_IL12\_12H\_CD4\_TCELL\_UP[GSEA]

|  || Dataset | filtered\_dataset.sample\_info.cls#WT\_versus\_NCAPD3\_MUT |
| Phenotype | sample\_info.cls#WT\_versus\_NCAPD3\_MUT |
| Upregulated in class | NCAPD3\_MUT |
| GeneSet | GSE17974\_CTRL\_VS\_ACT\_IL4\_AND\_ANTI\_IL12\_12H\_CD4\_TCELL\_UP |
| Enrichment Score (ES) | -0.49852994 |
| Normalized Enrichment Score (NES) | -2.751423 |
| Nominal p-value | 0.0 |
| FDR q-value | 0.0010500674 |
| FWER p-Value | 0.002 |
Table: GSEA Results Summary

  

Fig 1: Enrichment plot: GSE17974\_CTRL\_VS\_ACT\_IL4\_AND\_ANTI\_IL12\_12H\_CD4\_TCELL\_UP      
 Profile of the Running ES Score & Positions of GeneSet Members on the Rank Ordered List

  

| SYMBOL | TITLE | RANK IN GENE LIST | RANK METRIC SCORE | RUNNING ES | CORE ENRICHMENT || 1 | 134957 | STXBP5 | 170 | 0.709 | -0.0861 | No |
| 2 | 6426 | SRSF1 | 243 | 0.637 | -0.1047 | No |
| 3 | 130502 | TTC32 | 517 | 0.465 | -0.2785 | No |
| 4 | 400986 | ANKRD36C | 673 | 0.389 | -0.3706 | No |
| 5 | 6303 | SAT1 | 850 | -0.233 | -0.4862 | Yes |
| 6 | 200315 | APOBEC3A | 866 | -0.278 | -0.4824 | Yes |
| 7 | 6515 | SLC2A3 | 873 | -0.283 | -0.4719 | Yes |
| 8 | 50640 | PNPLA8 | 881 | -0.296 | -0.4613 | Yes |
| 9 | 220213 | OTUD1 | 889 | -0.304 | -0.4503 | Yes |
| 10 | 8848 | TSC22D1 | 899 | -0.324 | -0.4397 | Yes |
| 11 | 4616 | GADD45B | 904 | -0.330 | -0.4252 | Yes |
| 12 | 4209 | MEF2D | 910 | -0.337 | -0.4110 | Yes |
| 13 | 9314 | KLF4 | 972 | -0.389 | -0.4348 | Yes |
| 14 | 6239 | RREB1 | 974 | -0.391 | -0.4148 | Yes |
| 15 | 51646 | YPEL5 | 1014 | -0.413 | -0.4213 | Yes |
| 16 | 23764 | MAFF | 1039 | -0.436 | -0.4157 | Yes |
| 17 | 8609 | KLF7 | 1071 | -0.457 | -0.4141 | Yes |
| 18 | 54541 | DDIT4 | 1133 | -0.502 | -0.4319 | Yes |
| 19 | 388403 | YPEL2 | 1161 | -0.527 | -0.4237 | Yes |
| 20 | 665 | BNIP3L | 1185 | -0.556 | -0.4111 | Yes |
| 21 | 7128 | TNFAIP3 | 1198 | -0.573 | -0.3895 | Yes |
| 22 | 5795 | PTPRJ | 1210 | -0.586 | -0.3665 | Yes |
| 23 | 4189 | DNAJB9 | 1223 | -0.598 | -0.3437 | Yes |
| 24 | 2043 | EPHA4 | 1228 | -0.602 | -0.3147 | Yes |
| 25 | 54491 | FAM105A | 1241 | -0.619 | -0.2908 | Yes |
| 26 | 114907 | FBXO32 | 1248 | -0.625 | -0.2621 | Yes |
| 27 | 2355 | FOSL2 | 1262 | -0.648 | -0.2373 | Yes |
| 28 | 7994 | KAT6A | 1279 | -0.666 | -0.2137 | Yes |
| 29 | 57561 | ARRDC3 | 1324 | -0.744 | -0.2063 | Yes |
| 30 | 5997 | RGS2 | 1336 | -0.770 | -0.1736 | Yes |
| 31 | 84959 | UBASH3B | 1338 | -0.776 | -0.1333 | Yes |
| 32 | 133746 | JMY | 1350 | -0.793 | -0.0994 | Yes |
| 33 | 54463 | FAM134B | 1356 | -0.815 | -0.0600 | Yes |
| 34 | 10397 | NDRG1 | 1380 | -0.925 | -0.0278 | Yes |
| 35 | 6711 | SPTBN1 | 1381 | -0.925 | 0.0211 | Yes |
Table: GSEA details [plain text format]

  

Fig 2: GSE17974\_CTRL\_VS\_ACT\_IL4\_AND\_ANTI\_IL12\_12H\_CD4\_TCELL\_UP      
 Blue-Pink O' Gram in the Space of the Analyzed GeneSet

  

Fig 3: GSE17974\_CTRL\_VS\_ACT\_IL4\_AND\_ANTI\_IL12\_12H\_CD4\_TCELL\_UP: Random ES distribution      
 Gene set null distribution of ES for **GSE17974\_CTRL\_VS\_ACT\_IL4\_AND\_ANTI\_IL12\_12H\_CD4\_TCELL\_UP**
